# Keratoacanthoma Centrifugum Marginatum with Atypical Scar

**DOI:** 10.1155/2012/158158

**Published:** 2012-12-04

**Authors:** Falguni Nag, Projna Biswas, Joydeep Singha, Arghyaprasun Ghosh, Trupti V. Surana

**Affiliations:** Department of Dermatology, IPGMER & SSKM Hospital, kolkata 700020, India

## Abstract

Keratoacanthoma centrifugum marginatum (KCM) is a rare variant of keratoacanthoma (KA). It is characterized by a progressive peripheral expansion and central healing leaving atrophic scar. It is sometimes confused with squamous cell carcinoma (SCC) both clinically and histopathologically. We here report a case of KCM over the extensor aspect of the right forearm in a 57-year-old man with an abnormal looking scar.

## 1. Introduction

Keratoacanthoma (KA) is a rare benign epidermal tumour, usually diagnosed clinically, and histopathological examination confirms the diagnosis. Most lesions are classical solitary but few unusual rare variants are also described. Keratoacanthoma centrifugum marginatum (KCM) is a rare variant of solitary KA with a clinical course of centrifugal expansion and central restitution or scarring [1]. The cause is unknown but proposed association with UV radiation, chemical exposure, and viral infection has been reported [2]. We hereby report a case of KCM with atypical scar for its rarity and highlight its relationship with SCC.

## 2. Case Report

**Figure 1:**
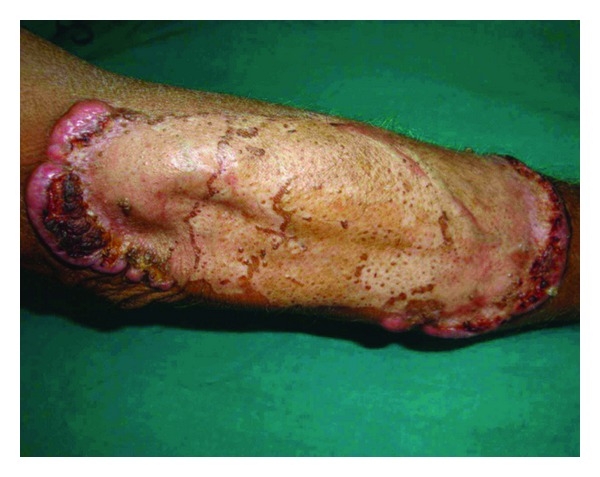
A plaque with nodular ulcerated margin and central scaring.

**Figure 2:**
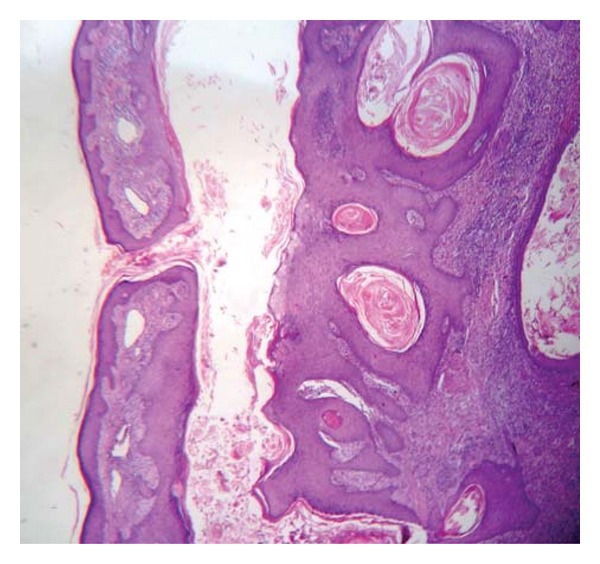
Photomicrograph showing invagination of epidermis and keratin filled crater at the centre [10x H&E].

**Figure 3:**
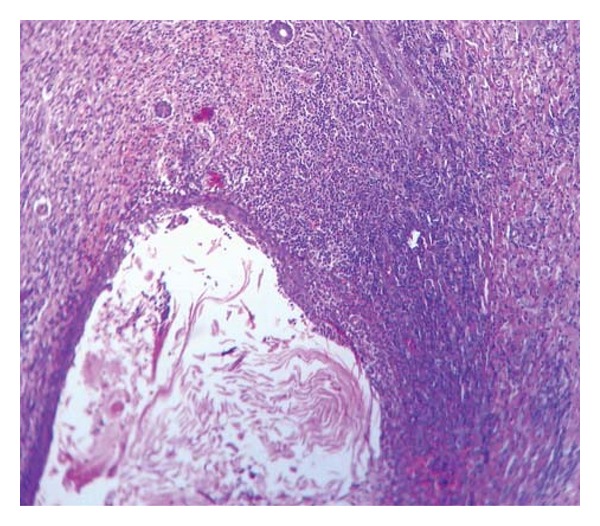
Photomicrograph showing dense mixed inflammatory cell infiltrate in the dermis immediately adjacent to the crater [100x H&E].

A 57-year-old male, a farmer by profession, presented in the dermatology OPD with a large plaque extending from below the right elbow onto the extensor aspect of the forearm. The lesion started as an asymptomatic nodule on a normal looking skin and attained its present state over the past 1 year by peripheral spreading and central healing. Nodular ulcerated margin was seen on the proximal and distal aspects in an arciform manner (Figure 1). The whitish intervening scar was traversed in its entire width by narrow strips of a normal looking skin. He was also treated with antitubercular medications from outside without any benefit. General survey, systemic examination, and routine laboratory investigations did not reveal any abnormality. Chest X-ray was normal. The Mantoux test and the screening for HIV were negative. A provisional diagnosis of KCM was made. Histopathological examination of a skin biopsy specimen from the raised margin revealed a huge pitcher like crater extending symmetrically across the upper dermis surrounded by epidermis on all sides (Figure 2). A keratin plug is seen at the bottom of the crater with a dense mixed inflammatory cell infiltrate in the dermis immediately adjacent to the crater (Figure 3). No cellular atypia or involvement of the deep dermis is seen. Based on the above clinical and histopathological findings diagnosis of KCM was made. The patient was referred to the plastic surgery department for excision and grafting. 

## 3. Discussion

Keratoacanthoma is a rapidly growing tumour with a tendency to spontaneous regression but it can recur if incompletely excised and if occurring over fingers, hands, lips, and pinnae [3]. There are several rare variants of KA like giant keratoacanthoma, KCM, multiple KA including generalized eruptive KA, and KA of unusual sites [2]. Solitary KA is the most common type with its three phases: proliferation, maturation, and spontaneous involution [4]. KCM is an uncommon type of solitary KA and characterized by progressive peripheral expansion with central healing leaving an atrophic scar [1]. It is mostly found over chronic sun exposed areas like lower legs and dorsa of hands. It was first described by Belisario in 1965 [5]. The lesion may reach up to 20 cm in diameter with no tendency to spontaneous regression [2], as was seen in our case. Although the exact cause of KA is unknown there is a distinct association with UV radiation, chemical exposure, and viruses, especially HPV [2]. Chronic sun exposure due to his profession might have played a role in our case. KCM should be differentiated from giant KA. Usually the latter is associated with underlying tissue destruction whereas KCM is not. The most important differential diagnosis of KA is squamous cell carcinoma (SCC). SCC develops from normal epidermal keratinocytes, but KA is derived from the supraseboglandular parts of hair follicles [6]. KA may rarely progress to SCC, and the two might coexist together in the same lesion [7]. Hodak and colleagues proposed that KA is a variant of SCC and can metastasize [8], while others did not find any full-proof relationship between the two [7]. In a fully developed lesion of KA a large irregular crater filled with keratin is seen at the centre, and the epidermis extends like a lip at the sides of the crater giving the lesion symmetry [7]. The epidermis proliferates and extends both upwards and downwards from the crater. The base of the lesion is regular and well demarcated in contrast to SCC and does not extend below the level of the sweat gland [9]. This is evident in our HPE also. Further, a thin well-demarcated stroma separates the proliferating epidermis from the dermis which is rarely invaded by proliferating cells. Atypical keratinocytes seen in SCC are rarely found, and the dense mixed inflammatory cell infiltrate in the adjacent dermis, as seen in our case, is not encountered in SCC. A progressive involution and fibrosis towards the centre of the lesion are particularly evident in KCM [7]. Although KCM is known to leave a central depressed scar while progressing, it had a particular look in our case in the form of a mildly raised whitish scar traversed by thin strands of a normal looking skin in its entirety. This could be explained by the small periods of inactivity during involution and regression when there was some repair from the periphery, followed by extension again. Lupus vulgaris, annular elastolytic giant cell granuloma, elastosis perforans serpiginosa, and SCC could be considered in the clinical differential diagnosis of KCM in its varying stages. Treatment options are not many including topical 5-fluorouracil, curettage and coagulation of the base, excision and suture, and radiotherapy [10]. 

We thought of reporting this case because KAs are rare while KCM with a peculiar looking scar is even rarer and also to highlight the simmering controversy of its relationship with SCC.

## 4. Conclusion

 KCM should be a differential diagnosis in skin lesion with central atypical scaring and peripheral spreading, and it should always be differentiated from SCC by histopathological examination.
